# Effectiveness and Safety of Laparoscopic Sleeve Gastrectomy for Weight Loss in Mild Obesity: Prospective Cohort Study with 3-Year Follow-up

**DOI:** 10.1007/s11695-022-05958-5

**Published:** 2022-02-24

**Authors:** Ahmed Elnabil-Mortada, Haitham M. Elmaleh, Roger Ackroyd, Rabbah A. Khaled

**Affiliations:** 1 Department of General Surgery, Faculty of Medicine, Ain Shams University, Cairo, Egypt; 2 Department of General Surgery, Sheffield Teaching Hospitals NHS Foundation Trust, Sheffield, UK

**Keywords:** Sleeve gastrectomy, Class I obesity, BMI 30–34.9, Mild obesity, Weight loss, Psychosocial impact of obesity

## Abstract

**Purpose:**

Patients with mild obesity especially in absence of associated medical problems (OAMP) are commonly managed by non-surgical approaches. Laparoscopic sleeve gastrectomy (LSG) has proved itself to be effective and it is now the most performed weight loss procedure. We aimed to study the effectiveness and safety of LSG for weight loss in mild obesity.

**Methods:**

A prospective cohort study. Group A; BMI (30–34.9 kg/m^2^), and group B; BMI ≥ 40 or BMI ≥ 35 with OAMP. Demographic data, perioperative complications, % excess weight loss (EWL), % total weight loss (TWL), nutritional profile, and evolution of OAMP were recorded and statistically analyzed.

**Results:**

A total of 250 patients, with 80 patients (32%) in group A, and 170 (68%) in group B. The majority were female. The mean preoperative weight, BMI, and excess weight were 90.1 ± 9.52, 32.7 ± 1.4, and 21.5 ± 4.9 in group A, and 129.88 ± 26.12, 47.8 ± 8.2, and 62.3 ± 23.6 kg in group B respectively. The low BMI group had significantly lower OAMP, with higher pre-LSG non-surgical procedures rate. Overall post-operative morbidity rate was significantly higher in group B. %TWL was significantly lower in low BMI group. Nutritional profile was within the normal range in both groups at 3-year follow-up.

**Conclusion:**

Laparoscopic sleeve gastrectomy is a safe and effective weight loss solution for mild obesity with better outcome than for higher BMI. Further studies are warranted to reconsider NIH’s statement for medicolegal aspects, and for matching the current changes in bariatric surgery practice, safety evidence, and patients’ demand.

**Graphical abstract:**

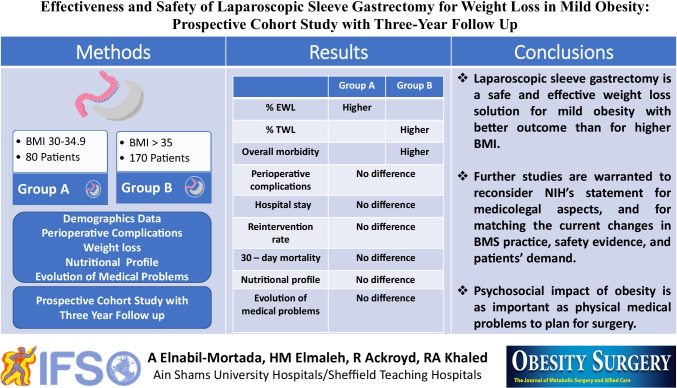

## Introduction

The current international medicolegal BMI cut-off for bariatric surgery in many countries is based on the National Institutes of Health (NIH) statement in 1991, with severe obesity (BMI > 40 kg/m^2^) or with less severe obesity (BMI 35–40 kg/m^2^) with associated medical problems [1].

The safety and effectiveness of the metabolic effect of bariatric and metabolic surgery (BMS) in patients with mild obesity have been thoroughly investigated in the literature especially in patients with T2DM and in Asian populations due to ethnic liability for OAMP. This has led different international and national BMS societies to recommend surgery in patients with class 1 obesity who do not achieve substantial and durable weight and OAMP improvement with non-surgical methods [2–9].

Although sleeve gastrectomy (SG) has remained the most performed bariatric procedure since 2014 (*N* = 386,096; 55.4%) according to an IFSO 2018 survey [10], few studies have been published for its effect on class 1 obesity especially in absence of associated medical problems.

Obesity is associated with a significant psychosocial burden, as many patients struggle with issues related to their mood, self-esteem, quality of life, and body image [11], which seems to be stronger for women than men [12]. It is believed to play an influential role in the decision to seek weight loss treatment even in the presence of significant weight-related health problems [13].

We aimed in our study to evaluate the effectiveness and safety of LSG as a weight loss solution for patients with class 1 obesity.

## Materials and Methods

This study was conducted in accordance with The Strengthening the Reporting of Observational Studies in Epidemiology (STROBE) statement [14]. The population, intervention, comparator, outcomes, and study design (PICOS) approach were used to identify the inclusion criteria (Table 1).

**Table 1:** PICOS criteria for the study

Parameter	Criteria
Population	Adults with obesity of BMI ≥ 30
Intervention	Laparoscopic sleeve gastrectomy
Comparator	Obesity with BMI 30–35, and BMI higher than 35
Outcomes	Perioperative complications, weight loss, nutritional profile, obesity associated medical problem (OAMP)
Study design	Prospective comparative cohort with 3-year follow-up

### Population

Two hundred and fifty adults aged between18 and 65 years diagnosed with obesity of BMI ≥ 30, who failed to achieve or maintain adequate weight loss with dietitian consultation, were included and divided into two groups. Group A represents the new proposed cut-off (patients with BMI 30–34.9 with/without OAMP and group B represents the current international cut-off (patients with BMI ≥ 40 or ≥ 35 with OAMP). Any patients with previous bariatric surgery, severe psychological disorders, documented severe gastroesophageal reflux disease (GORD) were excluded. No routine pre- or post-operative endoscopy has been done unless clinically indicated.

### *Intervention and Surgical Technique*

Laparoscopic sleeve gastrectomy was done between January 2015 and October 2017, in Ain Shams University Hospitals, Egypt. All patients were counselled carefully and signed informed consent which clearly mentioned the current BMI cut-off for surgery and group A gave their approval to share in this study understanding the current evidence.

Patients had to pay full cost of surgery in group A and pay 50–70% of the cost in group B as BMS in Egypt is not covered by insurance companies and partially funded in public health sector for current BMI cut-off. Only few cases are covered totally by charity. Accordingly, most of the patients were self-referral including the medical tourists or referred by primary care physicians or other medical specialists who were aware about our study.

We operated in the French position, using 3- 5 trocars. The first trocar was an optical trocar inserted 2 hand breadths below the xiphisternum and pneumoperitoneum was induced at a pressure of 15 mm Hg; the other trocars were inserted under vision. We cleared the greater curvature of the stomach from great omentum, starting at 4–6 cm from the pylorus up to the left crus, followed by firing sequences of staplers guided with a 34-Fr bougie, with no preference in the current available sealing and stapling devices. We did not place a drain and no regular post-operative contrast studies were performed unless clinically indicated. Patients started to drink and received a first prophylactic low molecular weight heparin dose 6 h post-operatively. Patients were discharged the following day unless clinically unwell.

A dietetic booklet was given to all patients, including the vitamin supplement requirement. Follow-up was at 1 week, then at 1, 3, 6, 12 months then yearly for 3 years. Consultations were either face to face or by email, WhatsApp, or phone call especially during COVID-19 pandemic. Patients were asked to do a full nutritional blood check-up including full blood count (FBC), iron study, B12, folic acid, Vit D, Ca, albumin, total protein yearly.

### Comparators

Group A, which represents new proposed cut-off for BMS (patients with BMI 30–34.9 with/without OAMP), was compared against group B, which represents current international cut-off (Patients with BMI ≥ 40 OR ≥ 35 with OAMP).

### Outcomes

Perioperative complications; weight loss parameters expressed as weight in kg, EWL%, and TWL%; nutritional profile; and evolution of OAMP were recorded and statistically compared between both groups annually for 3 years.

Ideal body weight was calculated based on a BMI of 25 kg/m^2^. Successful weight loss was defined as %EWL of ≥ 50% and %TWL of ≥ 20%.

### Study Design

Prospective cohort study with 3-year follow-up. The approval of the Ethics Committee in our hospital was obtained before the start of the study. The acquired data were entered in a data sheet (Microsoft Excel) and exported to a statistical program for social science (SPSS) for analysis between both groups.

## Results

### Demographic Characteristics of Patients

Table 2 summarizes the characteristics of the 250 patients in both groups. There was no significant difference in the mean age in both groups, with most of the patients being female. There was a statistical difference in preoperative weight, BMI, and excess weight between the groups. Thirty-two patients (40%) of low BMI had at least one or more of OAMP with a statistically significant lower percentage in comparison with 60% in higher BMI. Group A underwent at least one of the non-surgical interventions as intra-gastric balloon insertion, liposuction, or other procedure before LSG with statistically highly significance rate than group B.

**Table 2:** Patient’s demographics and preoperative data

Parameter	Group A (BMI:30–34.9)	Group B (BMI > 35)	*P* value
Total patient number	80	170	N/A
Age (mean ± SD)	35.83 ± 9.6	35.94 ± 10.28	0.93
Sex (F/M), female (%)	49/31 (61%)	125/45 (73.5%)	0.0457*
Pre-operative weight	90.1 ± 9.52	129.88 ± 26.12	< 0.0001**
Pre-operative BMI	32.70 ± 1.4	47.89 ± 8.21	< 0.0001**
Pre-operative excess weight	21.5 ± 4.97	62.38 ± 23.60	< 0.0001**
Pre-operative OAMP (%)	32/80 (40%)	102/170 (60%)	0.0032**
Previous endoscopic/aesthetic Procedures	60/80 (75%)	68/170 (40%)	< 0.0001**

^*^P-value: statistically significant **P-value: highly statistically significant

*OAMP* obesity-associated medical problems

### Perioperative Complications

Table 3 shows the intraoperative and post-operative complications between the groups. Overall morbidity rate was statistically significantly higher in the high BMI group, but there was no difference in peri-operative complications, hospital stay, reintervention rate, and 30-day mortality. There were four cases of intraoperative bleeding due to iatrogenic liver/splenic capsule tear which were controlled with compression and hemostatic agents. Two cases in group A (2.5%) returned to theatre, one underwent laparoscopic washout (LWO) for hemoperitoneum, while the other had laparoscopic washout with endoscopic stenting (Taewoong Niti-S™: Mega™ Esophageal Stent) for suture line leakage (SLL). Reintervention was necessary in five cases (2.9%) in group B, for which LWO was done for four cases; one with hemoperitoneum and three cases for SLL. In addition, two cases with SLL had oesophageal dilatation for stenosis (Reviewer 1, comment 6) and insertion of a mega stent, while third case was managed with an OVSCO clip (OTSC® GmbH, Tübingen, Germany) and Mega stent insertion. The fourth case with SLL in group B was male patient with BMI of 60, who presented with septic shock and peritonitis on D6 post-operatively, has undergone laparotomy with admission for intensive care unit for support of multiple organ failure, and eventually has passed away on day 10.

**Table 3:** Perioperative complications

Complications *N* (%)*	Group A (BMI:30–34.9) = 80	Group B (BMI > 35) = 170	*P* value
Intraoperative	0	4 (2.35%)	0.1678
Leakage	1 (1.25%)	4 (2.35%)	0.5628
Hemoperitoneum	1 (1.25%)	2 (1.17%)	0.9568
Wound infection	0	2 (1.17%)	0.3323
PE	0	1 (0.58%)	0.4092
Atelectasis/Pneumonia	0	4 (2.35%)	0.1678
Total morbidity	2 (2.5%)	17 (10%)	0.0372**
Hospital stay (days)***	1.06 ± 0.45	1.35 ± 2.34	0.2733
Reintervention	2 (2.5%)	5 (2.9%)	0.8577
30-day mortality	0	1 (0.58%)	0.4958

^*^N (%): number and percentage. **P-value: statistically difference. ***Days in mean ± Sd

### Weight Loss

Table 4 summarizes the pre-operative weight and BMI in both groups with the change over the study period with mean follow-up of 36 months. There was a highly significant pre-operative weight and BMI difference. %EWL was significantly higher in low BMI in comparison to %TWL which was higher in high BMI group. There was no difference in the number of patients who completed the follow-up between the groups.

**Table 4:** Weight loss change over the study period

		Follow-up time		
	Baseline	1 year	2 years	3 years
*Weight*				
Group A	90.1 ± 9.52	67.6 ± 6.25	65.8 ± 6.0	70.6 ± 4.0
Group B	129.88 ± 26.12*	81.72 ± 13.84*	78 ± 5.34*	83.0 ± 10.14*
*BMI*				
Group A	32.70 ± 1.4	24.61 ± 1.5	23.61 ± 2.5	25.4 ± 3.5
Group B	47.89 ± 8.21*	30 ± 4.1*	28.7 ± 9.1*	30.5 ± 4.1*
*% EWL*				
Group A	–	106.48 ± 21.50*	107 ± 16.50*	105.6 ± 12.50*
Group B	–	79.91 ± 12.20	80.2 ± 7.20	78.54 ± 8.0
*% TWL*				
Group A	–	24.8 ± 4.45	24.9 ± 3.5	23.9 ± 5.0
Group B	–	37 ± 5.72*	38 ± 1.72*	36 ± 5.0*
*% Follow-up*				
Group A	–	93.75% (75/80)	81.25% (65/80)	75% (60/80)
Group B	–	91.17%(155/170)	76.4%(130/170)	64.7%(110/170)

^*^P value < 0.0001: highly significant

### Nutritional Profile

Table 5 summarizes the nutritional values of both groups over the study period. The nutritional profile was assessed annually, which was generally within the accepted normal ranges for all variables, except for the mean vitamin D level at baseline, which was low in both groups. Vitamin D improved by year 1 and was maintained by the end of the study at 3-year follow-up, probably related to the effects of both supplementation and surgery.

**Table 5:** Nutritional status over the study period

		Follow-up time		
	Baseline	1 year	2 years	3 years
*Group A*				
Total protein (g/dL)	7.2 ± 0.4	7.2 ± 0.4	7.0 ± 0.3	7.1 ± 0.6
Albumin (g/dL)	4.0 ± 0.5	4.2 ± 0.2	4.3 ± 0.1	4.2 ± 0.3
Hemoglobin (g/dL)	14.1 ± 1.3	14.0 ± 0.8	14.3 ± 0.6	14.2 ± 0.4
Iron (ug/dL)	90 ± 42	103 ± 56	110 ± 25	107 ± 40
Ferritin (ng/mL)	210.0 ± 100	175 ± 110.4	195.6 135.4	200 ± 115
TIBC (ug/dL)	346.8 ± 60.7	296.5 ± 40	330.5 ± 21	335 ± 20
B12 (pg/mL)	473.8 ± 228	550.3 ± 142	541.3 ± 152	585.0 ± 612
Folic acid (ng/mL)	13.5 ± 6.9	19.5 ± 5.3	19.5 ± 4.1	18.5 ± 3.3
Vitamin D (ng/mL)	25.2 ± 8.1	33.1 ± 11.2*	35.6 ± 17.4*	40.5 ± 30.1*
Ca (mg/dL)	9.2 ± 0.4	9.1 ± 0.4	9.2 ± 0.1	9.4 ± 0.3
*Group B*				
Total protein (g/dL)	6.9 ± 0.1	7.0 ± 0.8	7.1 ± 0.3	7.0 ± 0.6
Albumin (g/dL)	3.9 ± 0.4	4.1 ± 0.2	4.0 ± 0.1	4.0 ± 0.3
Hemoglobin (g/dL)	13.3 ± 2.3	13.9 ± 0.8	14.1 ± 0.6	14.0 ± 0.5
Iron (ug/dL)	100 ± 30	102 ± 36	105 ± 35	106 ± 52
Ferritin (ng/mL)	220.0 ± 50	190 ± 80.4	197.6 ± 115.4	210 ± 120
TIBC (ug/dL)	326.8 ± 55.7	290.5 ± 30	340.5 ± 45	331 ± 20
B12 (pg/mL)	463.8 ± 110	560.3 ± 142	551.3 ± 251	545.0 ± 412
Folic acid (ng/mL)	11.5 ± 5.7	15.5 ± 5.3	17.5 ± 6.1	16.5 ± 5.3
Vitamin D (ng/mL)	22.2 ± 9.1	31.2 ± 20.1*	33.5 ± 15.8*	45.5 ± 29.7*
Ca (mg/dL)	9.5 ± 0.4	9.2 ± 0.2	9.6 ± 0.3	9.3 ± 0.1

^*^Change in baseline preoperative vitamin D level, and at 1, 2, 3 years

### Evolution of Obesity Associated Medical Problem (OAMP)

Table 6 summarizes pre-operative OAMP in both groups, with their evolution at 3-year follow-up, which is expressed as *Resolution/Improvement/No Change* (R/I/N). There was no statistically significant difference in resolution and improvement rate of OAMP in both groups.

**Table 6:** Preoperative obesity associated medical problem, and evolution rate at 3-year

Comorbidities	Group A (BMI:30–34.9) = 80		Group B (BMI > 35) = 170		*P* value
	Patient number (%)	Evolution rate* R/I/N	Patient number (%)	Evolution rate* R/I/N	
T2DM	20 (25%)	15/5/0	68 (40%)	47/21/0	0.6133**
Hypertension	8 (10%)	4/2/2	57 (33.5%)	30/20/7	0.6912**
Dyslipidemia	16 (20%)	10/4/2	91 (53.5%)	72/14/5	0.2588**
Hypothyroidism	5 (6.25%)	2/2/1	15 (8.8%)	7/5/3	0.7694**
Musculoskeletal	7 (8.75%)	4/2/1	85 (50%)	70/10/5	0.1085**
Sleep apnea	0	0	10 (5.88%)	7/3/0	N/A**
Fatty liver	13 (16.25%)	8/4/1	102 (60%)	70/20/12	0.6074**
Genitourinary	5 (6.25%)	3/1/1	30 (17.6%)	19/7/4	0.8892**

^*^Evolution rate: expressed as number of patients in order of R/I/N; resolution, improvement, no change. ** P value: no statistical difference in evolution of comorbidities

## Discussion

The statement of the National Institutes of Health in 1991 [1] has not been changed despite the tremendous changes in BMS practice over the last 30 years. The panel at that time decided the current cut-off (BMI > 40 or 35–40 kg/m^2^ with OAMP for bariatric surgery without the support of evidence-based data, but based on the balance between the risk and benefit of limited open bariatric surgery at that time) [3].

We believe that the main reason for not lowering the cut-off for surgery was the economic and national regularity conditions rather than the safety of the procedure, as already the action BMI cut points are reduced by 2.5 kg/m^2^ to BMI 27.5, 32.5, and 37.5 kg/m^2^ for Asian populations due to ethnic liability for obesity-associated medical problems [15]. In addition, many studies in the literature have proved the safety and effectiveness of metabolic surgery in patients with mild obesity especially in patients with T2DM [16–24]. A recent meta-analysis by Ji et al. showed the safety of BMS in general in Asian patients with type 2 diabetes and BMI < 30 kg/m^2^ [25], while Wang L concluded the same effect with laparoscopic sleeve gastrectomy on type 2 diabetes mellitus in patients with a body mass index (BMI) less than 30 kg/m^2^[26].

Based on the current evidence of the safety of metabolic surgery in class 1 obesity, combined with our national practice regulation, we planned our study to evaluate the effectiveness and safety of LSG as weight loss solution in patients with BMI 30–34.9, with or without OAMP. We looked at perioperative complications, weight loss change, nutritional profile, and evolution of OAMP, and compared the outcomes to patients with the current recommended BMI cut-off.

There was no significant difference between the mean age in both groups, with more than 50% were female patients in both groups. The younger mean age: 35 years old reflects the current effect of telemedicine and social media for increasing the awareness of BMS. All our study’s population failed to achieve or maintain the required weight by dietary consultations. In addition, 75% of group A underwent at least one of the non-surgical procedures with higher statistical difference than group B (40%) before attempting LSG, either intra-gastric balloon, liposuction, or abdominoplasty with disappointing results. This is reflecting the gap between the current recommended BMI cut-off and the patients’ demand. In the follow-up consultations, most of the group A patients have admitted that they would not have had those procedures done, if they have been offered the choice of LSG.

There was a statistically significant difference in preoperative weight, excess weight, and BMI 90.1 ± 9.52, 21.5 ± 4.97, and 32.70 ± 1.4 in group A compared to 129.88 ± 26.12, 62.38 ± 23.60, and 47.89 ± 8.21 in group B respectively with ideal BMI of 25 as the cut-off. Patients with low BMI have demonstrated statistically significant higher excess weight loss EWL% at 1, 2, and 3 years 106.48 ± 21.50, 107 ± 16.50, and 105.6 ± 12.50 respectively, as compared with the higher BMI group 79.91 ± 12.20, 80.2 ± 7.20, and 78.54 ± 8.0 respectively while the high BMI group showed significant higher %TWL over the 3-year period of the study.

We believe that %EWL is a better assessment of weight loss than %TWL as it is expected to be higher with high BMI patients as in our study, while %EWL indicates how much the patient succeeded to lose from the excess weight regardless of baseline weight; in addition, successful %TWL cut-off is not standardized in the literature, ranging from 10 to 25%, and is mainly used to assess non-surgical weight loss. In a recent systemic review by van Rijswijk, it was shown that %TWL is more accurate than EWL% [27].

EWL% in the low BMI group in our study was higher than other similar studies in the literature because we calculated using an ideal BMI of 25, while other publications studying class 1 obesity in Asian population calculated using an ideal BMI of 23. EWL% in our low BMI group will be 85% if measured using ideal BMI 23 which is comparable with our Asian literature.

Overall post-operative morbidity was significantly higher in the high BMI group, although there was no significant difference between both groups in individual peri-operative complications, hospital stay, 30-day mortality, and re-intervention rate (Table 3). Overall rate of SLL leakage in our study was 2% (5/250), stenosis 0.8% (2/250), morbidity 7.6% (19/250), and mortality 0.4% (1/250) which are comparable to published rate in the literature: 1–6%, 0.1–3.9%, 2–15%, and 0.14–0.5% respectively [28, 29].

All the values of the nutritional profile in both groups were within the normal range, apart from the mean vitamin D level at baseline, which was low in both groups. Vitamin D improved by 1 year and was maintained by the end of the study at 3-year follow-up, probably related to the effects of both supplementation and surgery. None of the patients had a post-operative BMI drop below 20 kg/m^2^.

We observed changes in physical OAMP in ≥ 80% in both groups without significant difference over 3-year follow-up. In our experience, while only 40% of group A had physical OAMP, and 60% in group B, the psychosocial impact of obesity was commonly present in both groups, with most of the patients being female. Depression, social anxiety disorders, and body image dissatisfaction seem to be stronger for women than men, perhaps because of society’s emphasis on thinness as a characteristic of female beauty [12, 30, 31].

Patients with mild obesity who failed to lose weight by non-surgical treatment are desperate for surgery. Early in our practice, we were reluctant to offer the surgery, which made their psychosocial impact worse and led them to engage in eating, to put weight to be candidate for surgery. Disordered eating is common among persons with obesity. Many patients presenting for weight loss treatment report that they engage in eating for emotional reasons [11].

Even in the presence of significant weight-related health problems, body image dissatisfaction is believed to play an influential role in the decision to seek weight loss treatment [13]. The substantial weight losses seen in the first 6 to 12 months after surgery are associated with dramatic changes in psychosocial status including depression, anxiety, quality of life, self-esteem, body image, and sexual function and often endure several years post-operatively [32].

Most BMS in Egypt is performed in the private sector as noticed worldwide, with rapidly expanding bariatric medical tourism industry with over 650 million people worldwide. According to a recent global survey by IFSO, we received 26.7% from the Gulf area especially Saudi Arabia. Medical tourists seek cheaper surgical options without long waiting lists and strict preoperative selection criteria in public health sector in their home country [33, 34].

The cost of BMS in many countries, especially the poorer ones, is not covered by the insurance companies or public health sector. So, in our experience, we do not believe that there was any great socioeconomic advantage that allowed for a good outcome for any of the groups over the other, as the patients in group B had to pay 50––70% of the cost even in the public hospital, while group A had to pay the full cost. Noun R et al. have published their results of 541 patients with mild obesity in Lebanon with excellent outcomes with a zero-fistula rate. Interestingly, all patients in their study had to self-fund the surgery [35].

Our results are like other studies which showed safety and effectiveness of LSG in class 1 obesity [22, 35–41], with better outcomes in the low BMI group. Like cancer surgery, where the best outcome is achieved at earlier stage or with neoadjuvant therapy, we believe that the outcomes of BMS are better when the surgery is offered in the early stages of obesity, and the current guidelines recommend preoperative weight loss with higher obesity stage to minimize the risks.

With increasing number of patients with mild obesity who present to surgery clinic due to the increased awareness, and widespread of BMS, we should consider offering them LSG after failure of other conservative methods. Otherwise, they will present later with higher obesity class due to worsening psychosocial impact and eating disorders, with increasing the risk of developing other OAMP.

Our study has some limitations and strengths. First, we did not include any data about the evolution of GORD although it is considered part of the long-term safety of LSG. GORD is complex multifactorial disease that requires objective tests and not only subjective questionnaire for accurate diagnosis. We did not offer LSG for severe GORD, patients with mild symptoms improved after weight loss and medical treatment, while patients with de novo severe GORD declined further investigations as they preferred conservative management to Roux-en-Y gastric bypass (RYGB). The second limitation is that we lumped group B together as one heterogenous group as we wanted to be more precise and focused on our study’s objective to compare the safety of LSG in the mild obesity group to the current established indications which were represented by group B, and subgroup analysis would not be of benefit to answer our question. The strength in our study is that it is one of few in the literature evaluating the role of LSG as weight loss solution in mild obesity.

In conclusion, laparoscopic sleeve gastrectomy is a safe and effective weight loss solution for mild obesity with better outcome than for higher BMI. Further studies are warranted to reconsider NIH’s statement for medicolegal aspects, and for matching the current changes in BMS practice, safety evidence, and patients’ demand. Psychosocial impact of obesity is as important as physical OAMP to plan for surgery.
